# AI-based localization and classification of skin disease with erythema

**DOI:** 10.1038/s41598-021-84593-z

**Published:** 2021-03-05

**Authors:** Ha Min Son, Wooho Jeon, Jinhyun Kim, Chan Yeong Heo, Hye Jin Yoon, Ji-Ung Park, Tai-Myoung Chung

**Affiliations:** 1 Department of Computer Science and Engineering, Sungkyunkwan University College of Computing, Sungkyunkwan University, 2044 Seobu-ro, Jangan-gu, Suwon, 16419 Republic of Korea; 2 Department of Plastic and Reconstructive Surgery, Seoul National University Boramae Hospital, Seoul National University College of Medicine, 5 Gil 20, Borame-Road, Dongjak-Gu, Seoul, 07061 Republic of Korea; 3 Department of Plastic and Reconstructive Surgery, Seoul National University Bundang Hospital, Seoul National University College of Medicine, Seongnam, 13619 Korea

**Keywords:** Diseases, Health care, Medical research, Signs and symptoms, Mathematics and computing

## Abstract

Although computer-aided diagnosis (CAD) is used to improve the quality of diagnosis in various medical fields such as mammography and colonography, it is not used in dermatology, where noninvasive screening tests are performed only with the naked eye, and avoidable inaccuracies may exist. This study shows that CAD may also be a viable option in dermatology by presenting a novel method to sequentially combine accurate segmentation and classification models. Given an image of the skin, we decompose the image to normalize and extract high-level features. Using a neural network-based segmentation model to create a segmented map of the image, we then cluster sections of abnormal skin and pass this information to a classification model. We classify each cluster into different common skin diseases using another neural network model. Our segmentation model achieves better performance compared to previous studies, and also achieves a near-perfect sensitivity score in unfavorable conditions. Our classification model is more accurate than a baseline model trained without segmentation, while also being able to classify multiple diseases within a single image. This improved performance may be sufficient to use CAD in the field of dermatology.

## Introduction

Computer-aided diagnosis (CAD) is a computer-based system that is used in the medical imaging field to aid healthcare workers in their diagnoses^1^. CAD has become a mainstream tool in several medical fields such as mammography and colonography^1,2^. However, in dermatology, although skin disease is a common disease, one in which early detection and classification is crucial for the successful treatment and recovery of patients, dermatologists perform most noninvasive screening tests only with the naked eye. This may result in avoidable diagnostic inaccuracies as a result of human error, as the detection of the disease can be easily overlooked. Furthermore, classification of a disease is difficult due to the strong similarities between common skin disease symptoms. Therefore, it would be beneficial to exploit the strengths of CAD using artificial intelligence techniques, in order to improve the accuracy of dermatology diagnosis. This paper shows that CAD may be a viable option in the field of dermatology using state-of-the-art deep learning models.

The segmentation and classification of skin diseases has been gaining attention in the field of artificial intelligence because of its promising results. Two of the more prominent approaches for skin disease segmentation and classification are clustering algorithms and support vector machines (SVMs). Clustering algorithms generally have the advantage of being flexible, easy to implement, with the ability to generalize features that have a similar statistical variance. Trabelsi et al.^3^ experimented with various clustering algorithms, such as fuzzy c-means, improved fuzzy c-means, and K-means, achieving approximately 83% true positive rates in segmenting a skin disease. Rajab et al.^4^ implemented an ISODATA clustering algorithm to find the optimal threshold for the segmentation of skin lesions. An inherent disadvantage of clustering a skin disease is its lack of robustness against noise. Clustering algorithms rely on the identification of a centroid that can generalize a cluster of data. Noisy data, or the presence of outliers, can significantly degrade the performance of these algorithms. Therefore, with noisy datasets, caused by images with different types of lighting, non-clustering algorithms may be preferred; however, Keke et al.^5^ implemented an improved version of the fuzzy clustering algorithm using the RGB, HSV, and LAB color spaces to create a model that is more robust to noisy data. SVMs have gained attention for their effectiveness in high-dimensional data and their capability to decipher “…subtle patterns in noisy and complex datasets”^6^. Lu et al.^7^ segmented erythema in the skin using the radial basis kernel function that allows SVMs to separate nonlinear hyperplanes. Sumithra et al.^8^ combined a linear SVM with a k-NN classifier to segment and classify five different classes of skin lesions. Maglogiannis et al.^9^ implemented a threshold on the RGB value for segmentation and used an SVM for classification. Although more robust than clustering algorithms, SVMs are more reliant on the preprocessing of data for feature extraction. Without preprocessing that allows a clear definition of hyperplanes, SVMs may also underperform.

Owing to the disadvantages of these traditional approaches, convolution neural networks (CNNs) have gained popularity because of their ability to extract high-level features with minimal preprocessing^10^. CNNs can expand the advantages of SVMs, such as robustness in noisy datasets without the need for optimal preprocessing, by capturing image context and extracting high-level features through down-sampling. CNNs can interpret the pixels of an image within its own image-level context, as opposed to viewing each pixel in a dataset-level context. However, although down-sampling allows CNNs to view an image in its own context, it degrades the resolution of the image. Although context is gained, the location of a target is lost through down-sampling. This is not a problem for classification, but causes some difficulty for segmentation, as both the context and location of the target are essential for optimal performance. To solve this, up-sampling is needed, which works in a manner opposite to that of down-sampling, in the sense that it increases the resolution of the image. While down-sampling takes a matrix and decreases it to a smaller feature map, up-sampling takes a feature map and increases it to a larger matrix. By learning to accurately create a higher-resolution image, CNNs can determine the location of the targets to segment. Thus, for segmentation, we use a combination of down-sampling and up-sampling, whereas for classification, we use only down-sampling. To further leverage the advantages of CNNs, skip-connections were introduced, which provided a solution to the degradation problem that occurs when CNN models become too large and complex. We implement skip-connections in both segmentation and classification models. In the segmentation model, blocks of equal feature numbers are connected between the down and up-sampling sections. In the classification model, these skip-connections exist in the form of inverted residual blocks. This allows our models to grow in complexity without any performance degradation.

In this paper, we present a method to sequentially combine two separate models to solve a larger problem. In the past, skin disease models have been applied to either segmentation or classification. In this study, we sequentially combine both models by using the output of a segmentation model as input to a classification model. In addition, although past studies of non-CNN segmentation models used innovative preprocessing methods, recent CNN developments have focused more on the architecture of the model than on the preprocessing of data. As such, we apply an innovative preprocessing method to the data of our CNN segmentation model. The methods described above lack the ability to localize and classify multiple diseases within one image; however, we have developed a method to address this problem. Our objective is two-fold. First, we show that CAD can be used in the field of dermatology. Second, we show that state-of-the-art models can be used with current computing power to solve a wider range of complex problems than previously imagined. We begin by explaining the results of our experimentation, followed by a discussion of our findings, a more detailed description of our methodology, and finally, the conclusions that can be drawn from our study.

## Results and discussion

**Figure 1:**
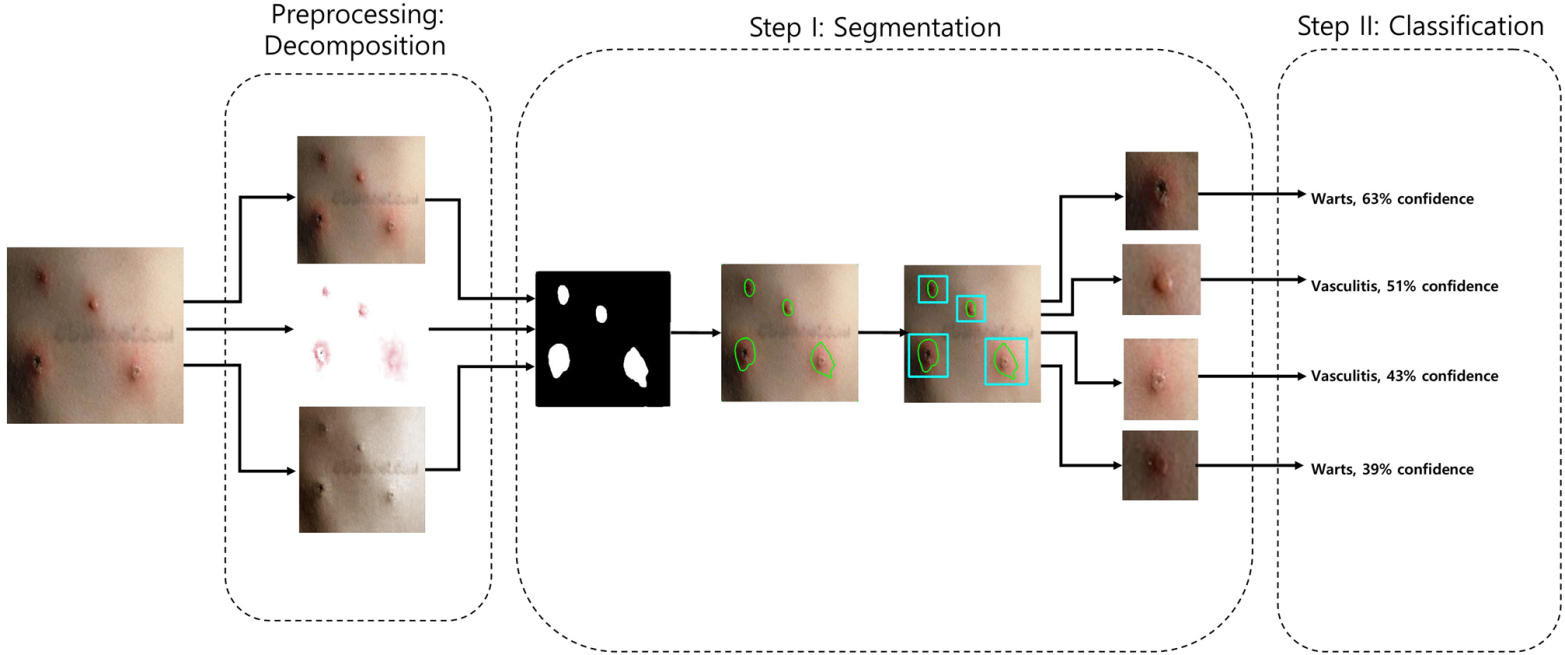
Schematic flow. From left to right, the original is first decomposed into hemoglobin and melanin images. All three images are input to the U-Net which outputs a black-and-white mask image. This mask image is used to draw contours each cluster. A convex hull algorithm is applied to crop each cluster. Each cluster is input to the EfficientNet, which generates a prediction alongside the confidence rate. An open- source implementation of the U-Net (v0.1.2) is available at: https://github.com/qubvel/segmentation_models.pytorch.

Figure 1 shows the schematic flow of our study. We started with the original image. We preprocessed this image by decomposing it into its hemoglobin and melanin constituents. These images were then input to the U-Net to generate the segmented output. We drew contours around each cluster and used a convex hull algorithm to draw rectangles around these clusters and crop them as individual images. These cropped images were used as input to the EfficientNet, which generated a prediction along with the confidence rate.

Table 1 shows the results of the test data for segmentation on our Dermnet dataset. The K-means clustering algorithm showed sub-optimal performance, owing to its limitations with noisy data. The SVM method showed a significant improvement in performance, that was attributed to the advantages of using SVMs to extract information from decomposition, rather than clustering algorithms. Even without the extra information, the U-Net trained without decomposition outperformed the previous two methods in terms of sensitivity. The U-Net model was also trained with decomposition and showed the highest sensitivity rate.

**Table 1:** Performance metrics for segmentation with dermnet images.

Method	Sensitivity	Specificity	Dice Coef	Hausdorff distance
K-means method	0.6148	0.6324	0.5165	10.487
SVM method	0.8200	0.8100	0.7123	8.138
U-Net method without decomposition	0.8953	0.7205	0.7215	8.153
U-Net method with decomposition	0.9589	0.7682	0.8126	7.165

In our results, we focused on the sensitivity metric because our objective was to assess the viability of using CAD with skin images. Although our U-Net model was not as good as the SVM model in terms of the specificity rate, it showed the best sensitivity rate, thus satisfying the objective of our study. In addition, we included the Dice coefficient and Hausdorff distance to demonstrate the performance of our methods with greater transparency. Our method showed clear improvements considering these alternative metrics. A major contributing factor^7^ to the underperformance of other methods is that performance of the SVM algorithm deteriorated when the images contained differences in lighting and shade. The K-means clustering method^3^ was also affected by the lighting and shade in the images. As our data had a significant mix of shade and lighting, the CNN was able to generalize the data better by learning to use the context of the image.

**Figure 2:**
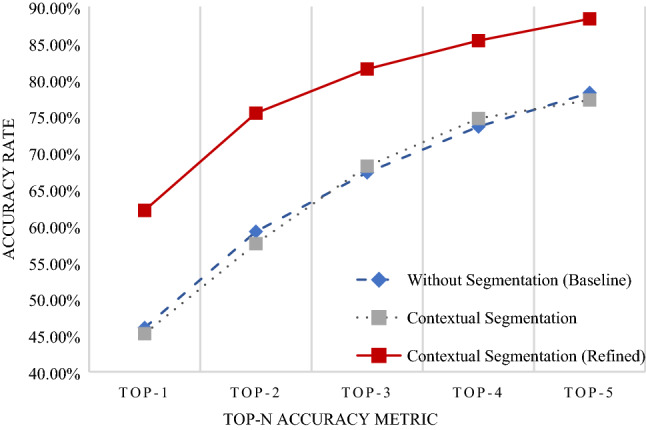
Accuracy rate for classification. The x-axis represents the Top-n accuracy metric, while the y-axis represents accuracy. The blue line is the accuracy of the model trained without segmentation. Images did not enter the U-Net before entering the EfficientNet. The gray line represents the accuracy of the model trained with segmentation. Images were segmented and cropped through the U-Net before entering the EfficientNet. The red line represents the accuracy of the model trained with segmentation and refined data. Images were segmented, cropped, and verified to ensure that segmentation had been done correctly before entering the EfficientNet. An open- source implementation of the EfficientNet (v0.7.0) is available at https://github.com/lukemelas/EfficientNet-PyTorch.

In any classification problem, it is important to set the baseline performance. We set our baseline to be the accuracy rate of the data without segmentation. The original image was input into the EfficientNet without going through the U-Net to determine the baseline accuracy rate. We compared this to the accuracy rate of the model trained to classify segmented images. Figure 2 shows the accuracy rates for the classification of our Dermnet dataset. We observed similar accuracy in the baseline model with and without contextual segmentation. The performance did not decrease when compared with the baseline. Thus, as we gained knowledge of the location of the disease without degrading the performance, we may say that the classification model was successfully implemented.

However, we were also aware that the accuracy may have decreased due to false positives caused by areas such as the lips, which have similar characteristics to erythema. Hence, a separate model was trained with refined data, where we went through each image and excluded those that were incorrectly segmented. This improved accuracy substantially, as shown in Figure 2. In addition, Table 2 shows additional metrics of the area under the curve (AUC), specificity, sensitivity, and F1-score. These values are weighted averages according to the number of data contained in each class. The AUC and specificity scores are high across all methods owing to the positive correlation of these metrics with the number of classes in a classification problem. Therefore, the more meaningful metrics in this dataset are the sensitivity and F1-score. The refined segmentation method demonstrated the highest performance considering these metrics, similar to the trend shown with the accuracy metric.

**Table 2:** Performance metrics for classification with dermnet images.

Method	AUC	Specificity	Sensitivity	F1-score
Without segmentation	0.8207	0.9642	0.4748	0.4092
Contextual segmentation	0.8104	0.9652	0.4185	0.3876
Refined contextual segmentation	0.8802	0.9513	0.6141	0.6079

**Figure 3:**
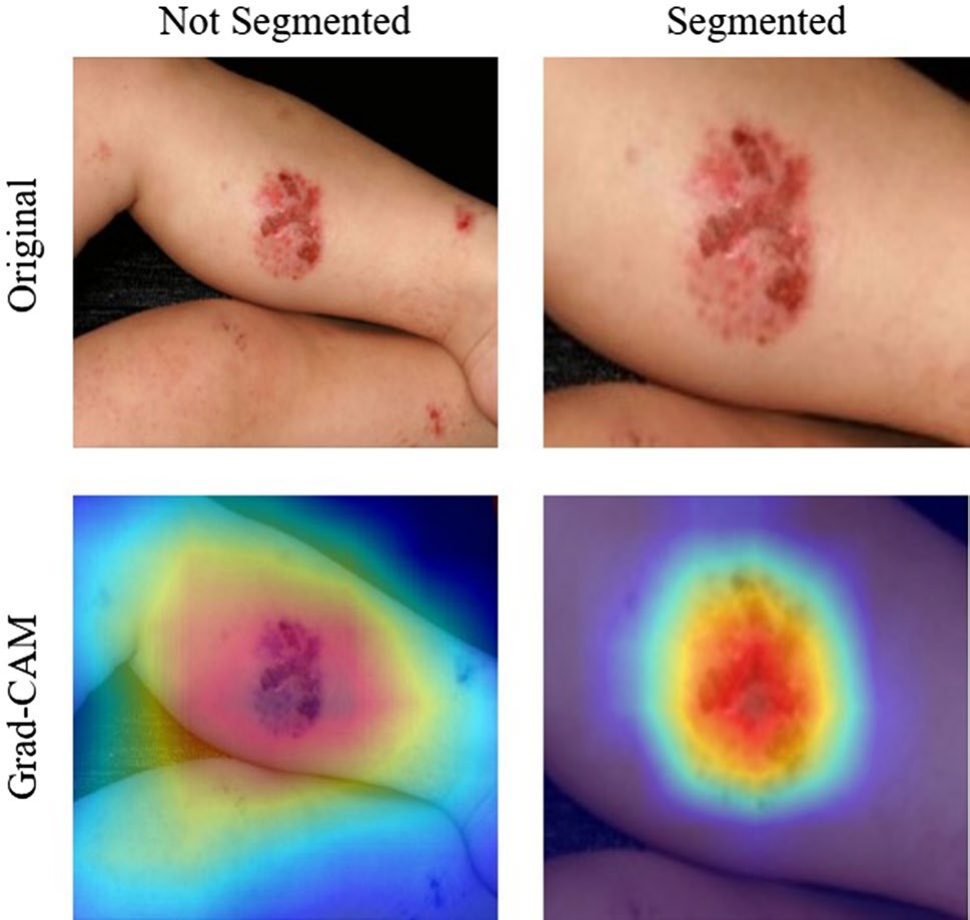
Grad-CAM results for unsegmented and segmented images in our Dermnet dataset. The top row shows the original input images. The left image shows the unsegmented image and the right image shows the segmented image. The bottom row shows the result of Grad-CAM^11^. The left image of Grad-CAM for the unsegmented image shows that the EfficientNet model focused on a larger surface other than erythema. The right image of Grad-CAM for the segmented image shows than that the EfficientNet model correctly focused mostly on erythema.

This was a result of an improved performance when there is a smaller area to search for the disease. Because we segmented only the abnormal areas of the skin, the EfficientNet model showed better performance compared to images with a larger ratio of normal skin. Thus, we can learn about the location of the disease that is present in an image and improve performance by training a CNN model to focus on particular subsections of the images. Figure 3 shows a visual representation of this claim using an implementation of the Grad-CAM method^11^. Activation, which is the intensity with which a model focuses on an area, is represented on a rainbow colormap. Red represents areas of highest activation, while violet represents areas of lowest activation. When trained with unsegmented data, our model focused on an area larger than that of abnormal skin. The area of activation was highest around the erythema, although there were other areas of high activation. In these cases, the model utilized the shapes of body parts for classification. This decreases performance because skin disease can appear in virtually any part of body and there is a lack of data required to form an association between the probability of a skin disease based on the body part. When trained with contextually segmented data, however, our model correctly focused only on erythema. The area of activation was highest around the erythema, while areas of low activation were demonstrated elsewhere. Not only does this add validity to our reported results, but this is also a justification for the inclusion of the segmentation phase before the classification phase because there were clear improvements in all metrics regarding the use of the U-Net before the EfficientNet.

Table 3 shows the results of the test data for segmentation on our three independent dermatoscopic image datasets—ISIC2016^12^, ISIC2017^13^, and HAM10000^14^. These datasets are some of the few publicly available datasets that had segmentation maps and classification labels. We use these datasets to verify our methods with data from independent sources. One major difference with the dermatoscopic image datasets is that they are obtained using a special dermatoscopic device. This eliminates noise in the form of background and non-skin areas, in addition to limiting the number of disease and fixing the location of skin disease within an image. This was shown to decrease the significance of our method.

**Table 3:** Performance metrics for segmentation with dermatoscopic datasets.

Method	Sensitivity	Specificity	Dice Coef	Hausdorff distance
**ISIC2016**				
K-means method	0.5422	0.8249	0.5439	9.960
SVM method	0.7229	0.8602	0.6939	8.243
U-Net method without decomposition	**0.9708**	0.9175	0.9060	5.085
U-Net method with decomposition	0.9562	**0.9422**	**0.9198**	**4.764**
**ISIC2017**				
K-means method	0.5709	0.7734	0.4926	10.567
SVM method	0.7650	0.7576	0.5967	9.388
U-Net method without decomposition	0.8971	0.8969	0.8188	5.392
U-Net method with decomposition	0.9043	0.9076	0.8199	5.338
**HAM 10,000**				
K-means method	0.5500	0.9300	0.6381	6.807
SVM method	0.7256	0.8389	0.6674	8.381
U-Net method without decomposition	0.9542	**0.9530**	0.9121	4.683
U-Net method with decomposition	**0.9569**	0.9504	**0.9166**	**4.621**

With the ISIC2016 and ISIC2017 datasets, the performance of the less-complex K-means clustering algorithm and SVM method showed similar trends to that of our Dermnet dataset. The performance was sub-optimal, owing to the noise present in the form of varying skin and lesion colors. With the HAM10000 dataset, however, the K-means clustering algorithm outperformed the SVM method in terms of the specificity and Hausdorff distance. This performance is a result of a more statistically similar training and testing set, as they were user-defined and created after stratifying the labels. Regardless of this, the less complex methods showed sub-optimal performances with all datasets.

Across all three datasets, the U-Net models outperformed previous models in all metrics. One interesting tendency is the small performance discrepancy between the U-Net models with and without decomposition. The U-Net model without decomposition occasionally outperformed the U-Net with decomposition. This was attributed to the skin lesion being mostly fixed at the center of the image. The hemoglobin and melanin constituents aid the U-Net model to ignore areas of non-skin and to focus on areas of skin with abnormal intensities. Therefore, this did not add significant information.

Table 4 shows the results of the test data for classification on the three dermatoscopic image datasets. With the ISIC2016 dataset, the *Without Segmentation* method showed the highest performance in all metrics. With the ISIC2017 dataset, the *Refined Contextual Segmentation* method showed the highest performance by a minimal margin. With the HAM10000 dataset, the *Without Segmentation* method showed the highest performance in all but one category. In short, with dermatoscopic images, models trained without segmentation learned to generalize skin lesions most effectively.

**Table 4:** Performance metrics for classification with dermatoscopic datasets.

Method	AUC	Specificity	Sensitivity	F1-score
**ISIC2016**				
Without segmentation	**0.765**	**0.726**	**0.860**	**0.864**
Contextual segmentation	0.719	0.641	0.826	0.833
Refined contextual segmentation	0.727	0.698	0.844	0.845
**ISIC2017**				
Without segmentation	**0.790**	0.741	0.761	0.740
Contextual segmentation	0.750	0.744	0.726	0.723
Refined contextual segmentation	0.774	**0.785**	**0.766**	**0.762**
**HAM 10,000**				
Without segmentation	**0.891**	**0.933**	0.866	**0.871**
Contextual segmentation	0.831	0.884	0.825	0.810
Refined contextual segmentation	0.871	0.919	**0.873**	0.866

**Figure 4:**
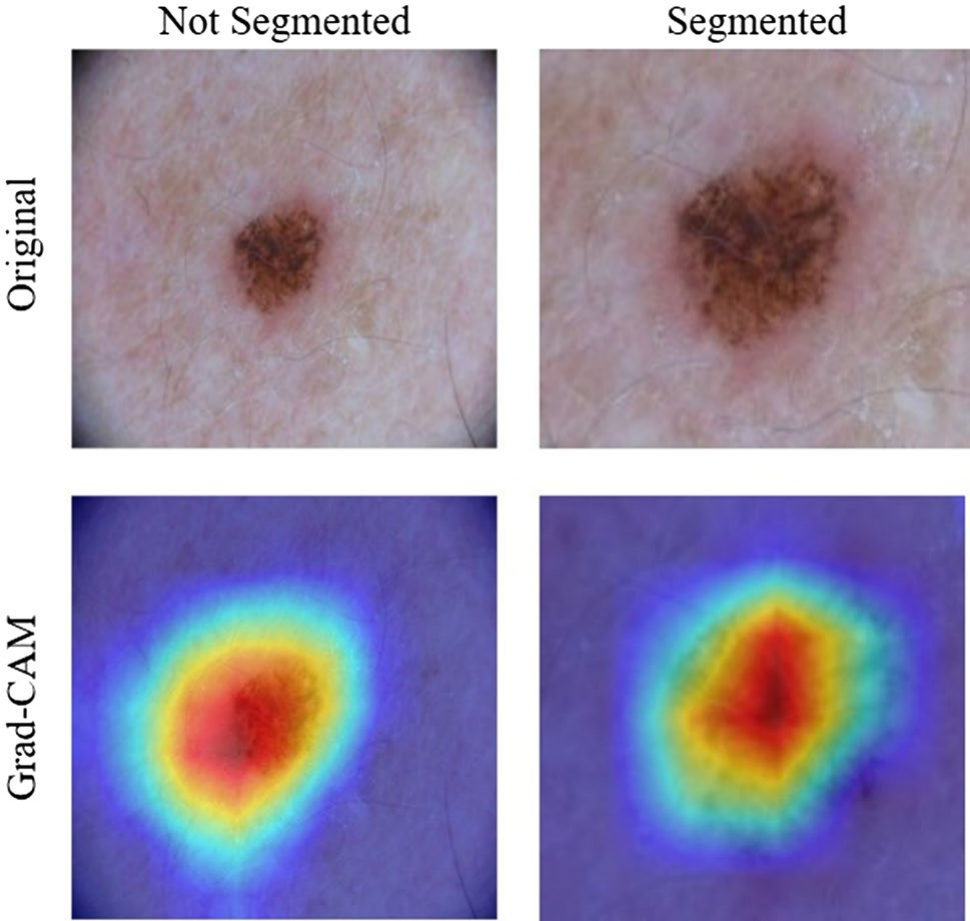
Grad-CAM results for unsegmented and segmented images in the ISIC2017 dataset. For both images of Grad-CAM, the EfficientNet model correctly focused mostly on erythema.

This was a result of an improved performance when the location of the skin lesion is mostly fixed. The segmentation phase aids models to ignore areas of normal skin and to focus on areas of disease. With dermatoscopic images, this information is insignificant, as the location of the disease is static. Figure 4 shows a visual representation of this. The Grad-CAM images show that with both non-segmented and segmented images, the models correctly focused on the skin disease. Because of this, the segmentation phase only decreased the resolution of the image without providing useful information, thus decreasing the performance of the model.

The main contribution of our study is researching the viability of CAD in the field of dermatology. This is achieved through the increase in the classification performance of skin disease images, owing to the increase in performance of segmentation. However, our model is most effective with camera images of skin diseases with erythema, which is a limitation of our study. We chose to focus on camera images and erythema because these images are very accessible, and erythema is one of the most common symptoms of skin disease. In addition, currently we only classify diseases into 18 categories due to the limitations of the data. In the future, we plan to create a more comprehensive skin disease classification model, and this seems to be viable if enough data can be obtained. In addition, we plan to work on a method to help dermatologists with time-series analysis of patients. This seems viable with the accumulation of data through CAD.

## Analysis methodology

**Figure 5:**
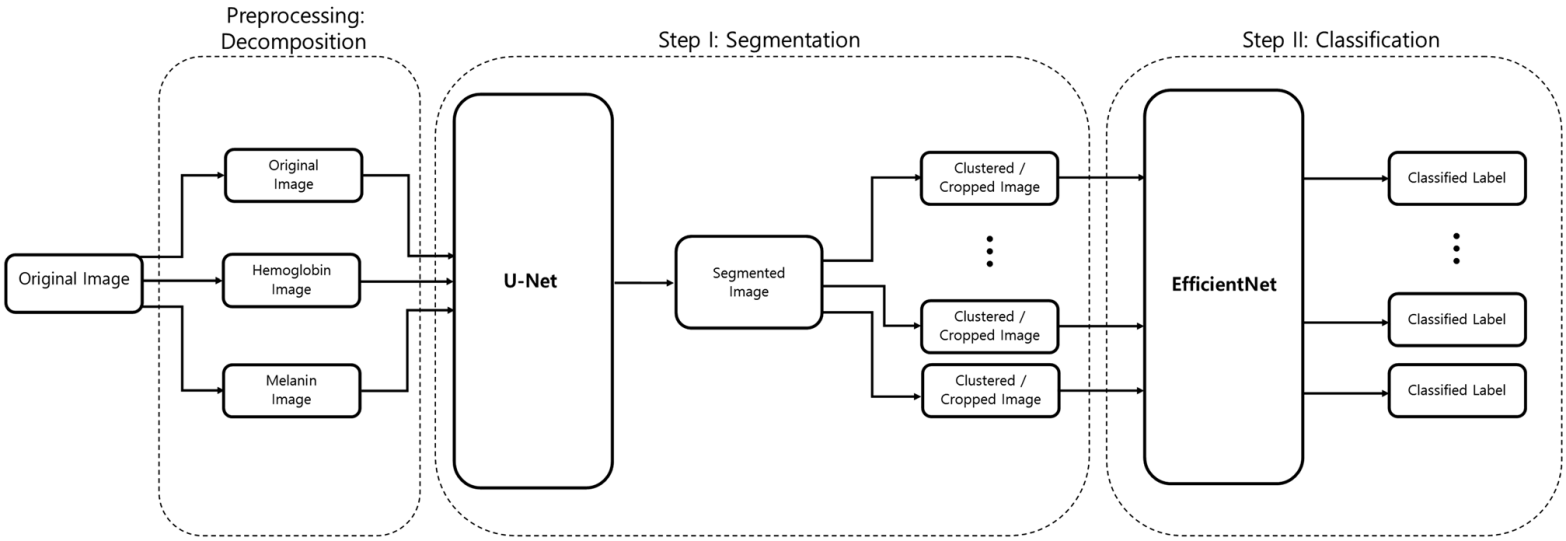
Two-phase analysis model. The original image primarily enters a preprocessing stage, where normalization and decomposition occur. Afterwards, the first step is segmentation, where cluster of abnormal skin are segmented and cropped. The second step is classification, where each cluster is classified into its corresponding class.

Our 2-phase analysis model for localization and classification is shown via the pseudocode in Algorithm 1 and visually in Fig. 5. We decomposed the original image into its hemoglobin and melanin constituents using preprocessing, to help our model extract valuable information from data that would have been otherwise unavailable. We provide these images as input to our segmentation model, the U-Net, which generated a segmented image. This segmented image was then analyzed for clusters, which were subsequently cropped and input to our classification model, the EfficientNet, which then produced a classified label, thus completing our analysis model.

The data for training and testing were obtained from Dermnet NZ, an archive of skin disease information launched and maintained by a group of dermatologists from New Zealand. The site provides open source images with labels. We selected 18 top-level categories (Table 5) each of which included enough data, besides including erythema as one of its common symptoms. Using a web crawler, we gathered a total of 15,851 images. Among the images obtained through Dermnet, the erythema of 100 images was masked by dermatologists, to be used as a ground truth. For segmentation, 60 images were used for training, and 40 images were used for testing. For classification, 13,473 images were used for training, and 2,378 images were used for testing. In addition, the test set for classification was split before segmentation cropping to prevent the subsections of one image from appearing in both the training and testing sets. Table 6 shows the distribution of data in greater detail. We chose the 100 images for segmentation in a balanced manner from each class, to minimize any bias that could occur during the classification phase.

**Table 5:** Categories for classification.

Top-level categories		
1. Acne and Rosacea 2. Actinic keratosis 3. Atopic dermatitis 4. Bullous disease 5. Cellulitis 6. Contact dermatitis	7. Eczema 8. Exanthems 9. Fungal infections 10. Herpes 11. Light chain disease 12. Lupus erythematosus	13. Psoriasis 14. Scabies 15. Systemic disease 16. Urticaria 17. Vasculitis 18. Viral infections

**Table 6:** Distribution of data in dermnet dataset.

Dataset: Dermnet	Number of data					
	Segmentation			Classification		
Class	Train	Test	Total	Train	Test	Total
Acne and Rosacea	4	2	6	746	131	877
Actinic keratosis	4	2	6	1193	181	1374
Atopic dermatitis	3	2	5	642	120	762
Bullous disease	3	2	5	393	92	485
Cellulitis	3	2	5	223	73	296
Contact dermatitis	3	2	5	231	74	305
Eczema	4	3	7	1667	234	1901
Exanthems	3	2	5	354	87	441
Fungal infections	4	3	7	1601	227	1828
Herpes	3	2	5	397	94	491
Light chain disease	3	2	5	538	117	655
Lupus erythematosus	3	2	5	371	90	461
Psoriasis	4	3	7	2044	275	2319
Scabies	3	2	5	448	98	546
Systemic disease	3	2	5	633	119	752
Urticaria	3	2	5	138	63	201
Vasculitis	3	2	5	411	94	505
Viral infections	4	3	7	1443	209	1652
Total	60	40	100	13,473	2378	15,851

One of the significant merits of the Dermnet dataset is that it was created and is maintained by a diverse group of dermatologists. The images in each top-level category are independent as they are images of different patients at distinct locations taken with varying devices. This is evident in the diverse resolutions, lighting, and aspect ratios of the images. Regardless, it would be optimal to possess a similar dataset from an entirely separate association to truly validate the performance of our model. However, as there are strict regulations regarding the use of data in our private institutions, we utilize publicly available datasets. These datasets were chosen based on the availability of both a segmentation map and a classification label.

ISIC2016^12^, ISIC2017^13^, and HAM10000^14^ are datasets that have been used in previous AI competitions. They were provided as challenges for both segmentation and classification, and they therefore possess segmentation maps and classification labels. Table 7 shows a detailed distribution of these datasets. As the ISIC2016 and ISIC2017 datasets also provided a separate test dataset, these datasets were preserved and used for testing. For the HAM10000 dataset, we stratified the dataset according to the classification label, and created a balanced 50% split between the train and test data. There is no separate segmentation dataset, as each image contained a segmentation map. Therefore, all images are used in the training and testing for both segmentation and classification.

**Table 7:** Distribution of data in dermatoscopic datasets.

Class	Number of data		
	Train	Test	Total
**Dataset: ISIC 2016**			
Benign	727	303	1030
Malignant	173	75	248
Total	900	378	1278
**Dataset: ISIC 2017**			
Benign	1372	393	1843
Melanoma	374	117	386
Seborrheic keratosis	254	90	521
Total	2000	600	2750
**Dataset: HAM 10000**			
Actinic keratosis	164	163	327
Basal cell carcinoma	257	257	514
Benign	549	550	1099
Dermatofibroma	58	57	115
Melanoma	556	557	1113
Melanocytic nevi	3352	3353	6705
Vascular lesion	71	71	142
Total	5007	5008	10,015

There is one significant difference between these datasets and our Dermnet dataset. The images in these datasets were obtained with a special dermatoscopic device. These devices create high-resolution images with the skin disease located near the center. Therefore, these devices create images similar to the Dermnet dataset images after our segmentation phase. Thus, it is doubtful that our method will demonstrate an improved performance with the dermatoscopic images.

For all datasets, the testing dataset is unused for validation until the end of training. This is done to verify that our models learn to generalize unseen images. We take a three-fold cross-validation approach with training data for validation during training. We generate three replicas of each dataset and create a unique 90-to-10 training and validation set. With each replica, we use a grid search algorithm to test different combinations of hyperparameters. Lastly, we train our model using the entire training set and select our hyperparameters based on the cross-validation stage. Training and testing were performed on a single GTX Titan V and four Intel Xeon Gold 5115 processors. We now explain each section of our analysis model in more detail.

**Table:** 

Algorithm 1 AnalyzeSkin
1: **procedure** SEGMENT(*x*)
2: *h, m* = DECOMPOSE(*x*)
3: *mask* = U-NET([*x, h, m*]) 4: CLASSIFY(*mask*)
5: **end procedure**
6: **procedure** CLASSIFY(*mask*)
7: *clusters* = FINDCLUSTERS(*mask*)
8: **for** *cluster* **in** *clusters* **do**
9: *cluster* = FIXRATIO(*cluster*)
10: *cluster* = RESIZE(*cluster*)
11: *class* = EFFICIENTNET(*cluster*) 12*. top_prediction* = GETHIGHESTCONFIDENCE(*class*)
12: print(*top_prediction*)
13: **end for**
14: **end procedure**

## Preprocessing: decomposition

**Figure 6:**
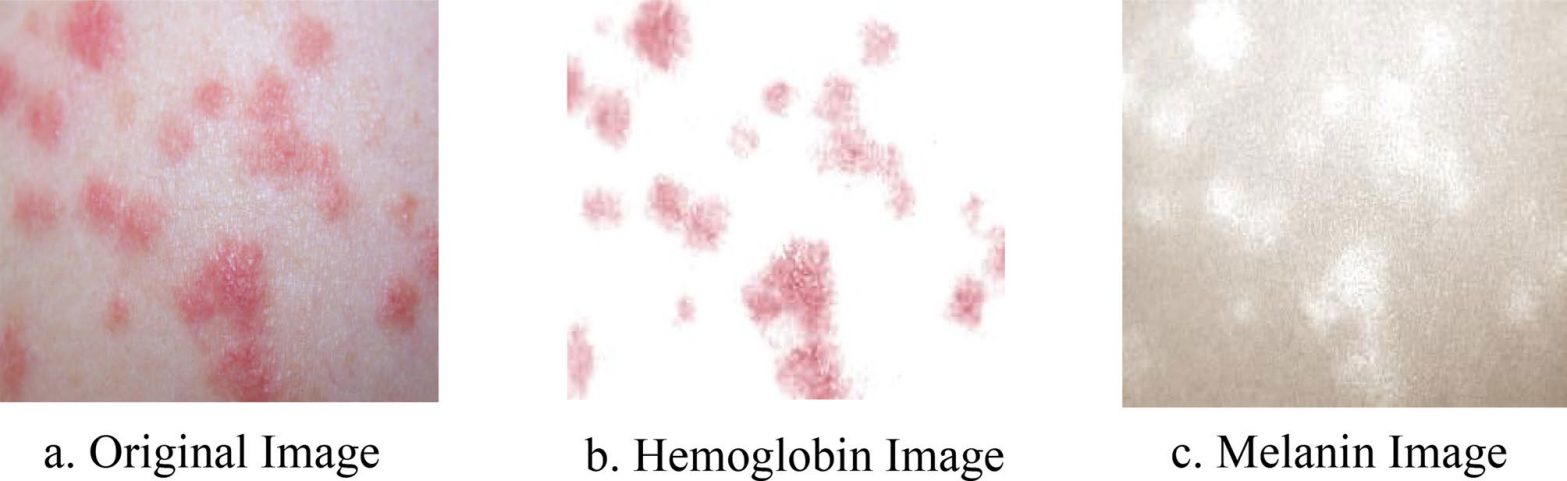
Decomposed result of skin. The original image is decomposed into its hemoglobin and melanin constituents through ICA.

The main constituents of the skin that are visible to humans are melanin and hemoglobin. These constituents provide valuable information for the segmentation of abnormal skin. To ensure that our model can learn to use these features, we used independent component analysis (ICA) to extract the melanin and hemoglobin constituents^7,15,16^. Assuming that these components are linearly separable, the separated linear vectors can be represented by the following formula^7^:

$${L}_{x,y}= {d}^{m}{q}_{x,y}^{m}+{d}^{h}{q}_{x,y}^{h}+ \Delta$$

where ${d}^{m}$ and ${d}^{h}$ represent the density vectors of melanin and hemoglobin, respectively, ${q}_{x,y}^{m}$ and ${q}_{x,y}^{h}$ represent the quantity of these components, and $\Delta$ represents values that are caused by other colors. As shown in^7^, by applying ICA, we can decompose skin as

$$\left[{q}_{x,y}^{m},{q}_{x,y}^{h}\right]= {\stackrel{-}{D}}^{-1}{L}_{\left(x,y\right)}-E$$

$$E= {min}_{x,y}\left({\stackrel{-}{D}}^{-1}{L}_{\left(x,y\right)}\right)$$

$${I}_{x,y}=exp(-{{L}^{^{\prime}}}_{x,y})$$

where $\stackrel{-}{D}$ represents the estimated values of ${d}^{m}$ and ${d}^{h}$, and ${I}_{x,y}$ represents the decomposed result. Figure 6 shows an example of one of these decompositions.

## Segmentation

**Figure 7:**
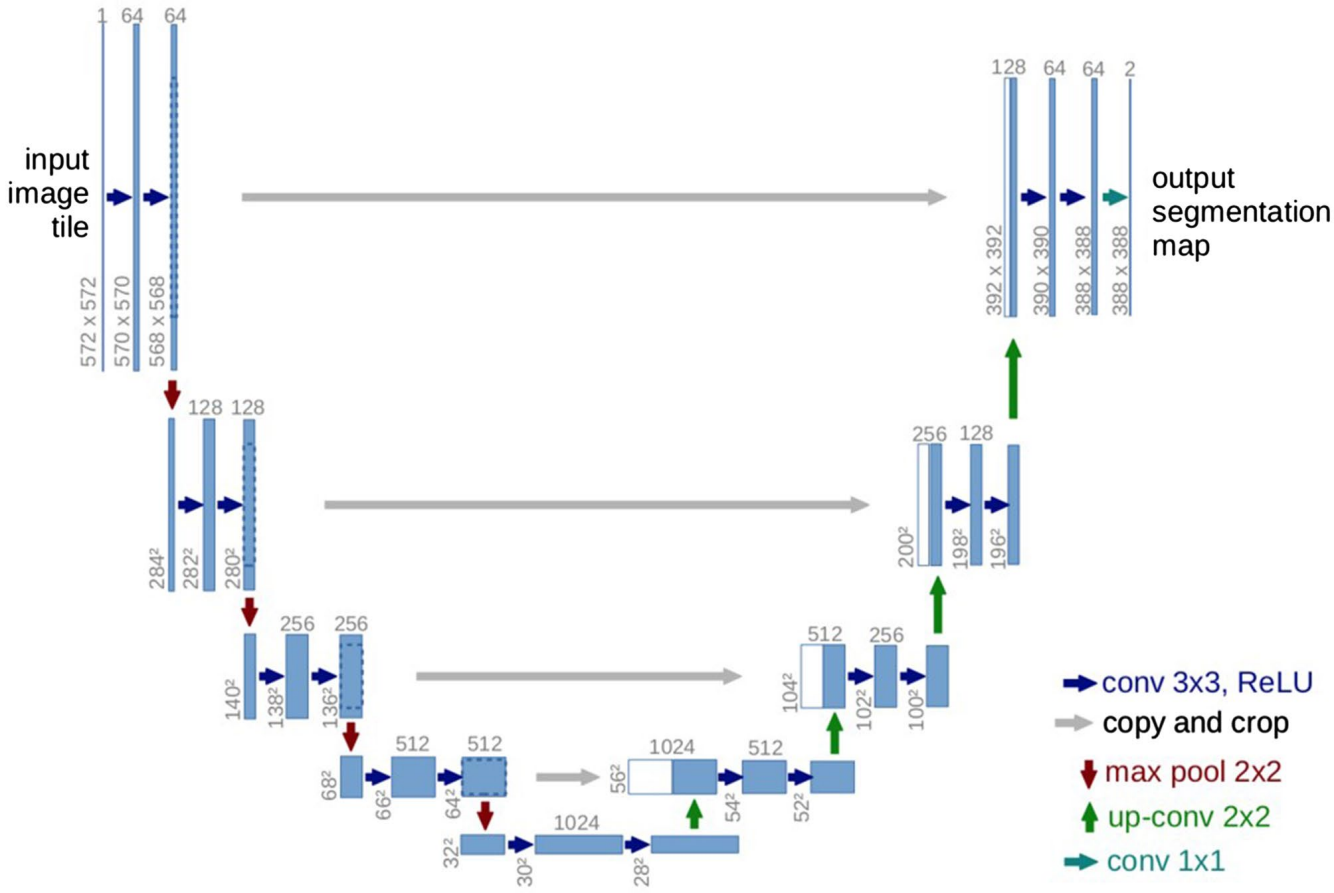
U-Net architecture. A fully CNN network, comprised of down-sampling, up-sampling, and skip connections^17^.

The U-Net^17^, as shown in Fig. 7, is an architecture created by CNNs, that has attracted attention for accurate biomedical image segmentation through the combination of down-sampling, up-sampling, and skip connections. Its name is attributed to the shape of its architecture, the first half of the ‘U’ representing down-sampling. Here, the context and key features of the input images are gained at the cost of a decrease in resolution. The second half of the ‘U’ represents up-sampling. Here, the resolution is increased to gain knowledge of the location of the target segment. To combat degradation due to the complexity of the model, skip connections are added to each up-sampling block.

Although in the original paper^17^, the resolutions of input and output were different, that is, 572 × 572 and 388 × 388 pixels, respectively, we chose to keep our input and output resolution consistent at 304 × 304 pixels. This was done because the images in our dataset were not large enough to warrant the tiling strategy required for extremely large images. Thus, zero-padding allowed us to keep the input and output resolutions consistent, thereby allowing the retention of information present on the border of our images.

**Figure 8:**
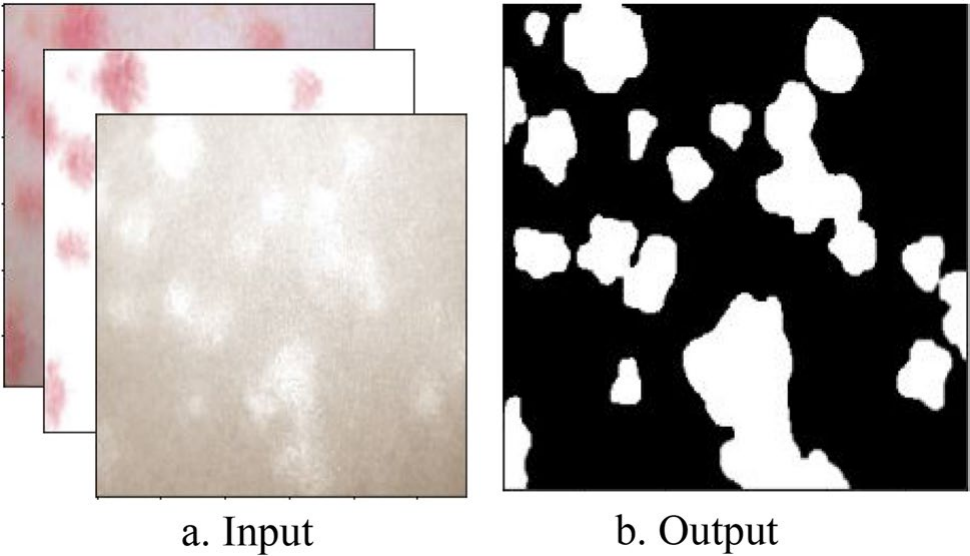
Input and Output of the U-Net. The inputs of the U-Net are the original, hemoglobin, and melanin images obtained from the preprocessing step. The output of the U-Net is a single masked image.

**Figure 9:**
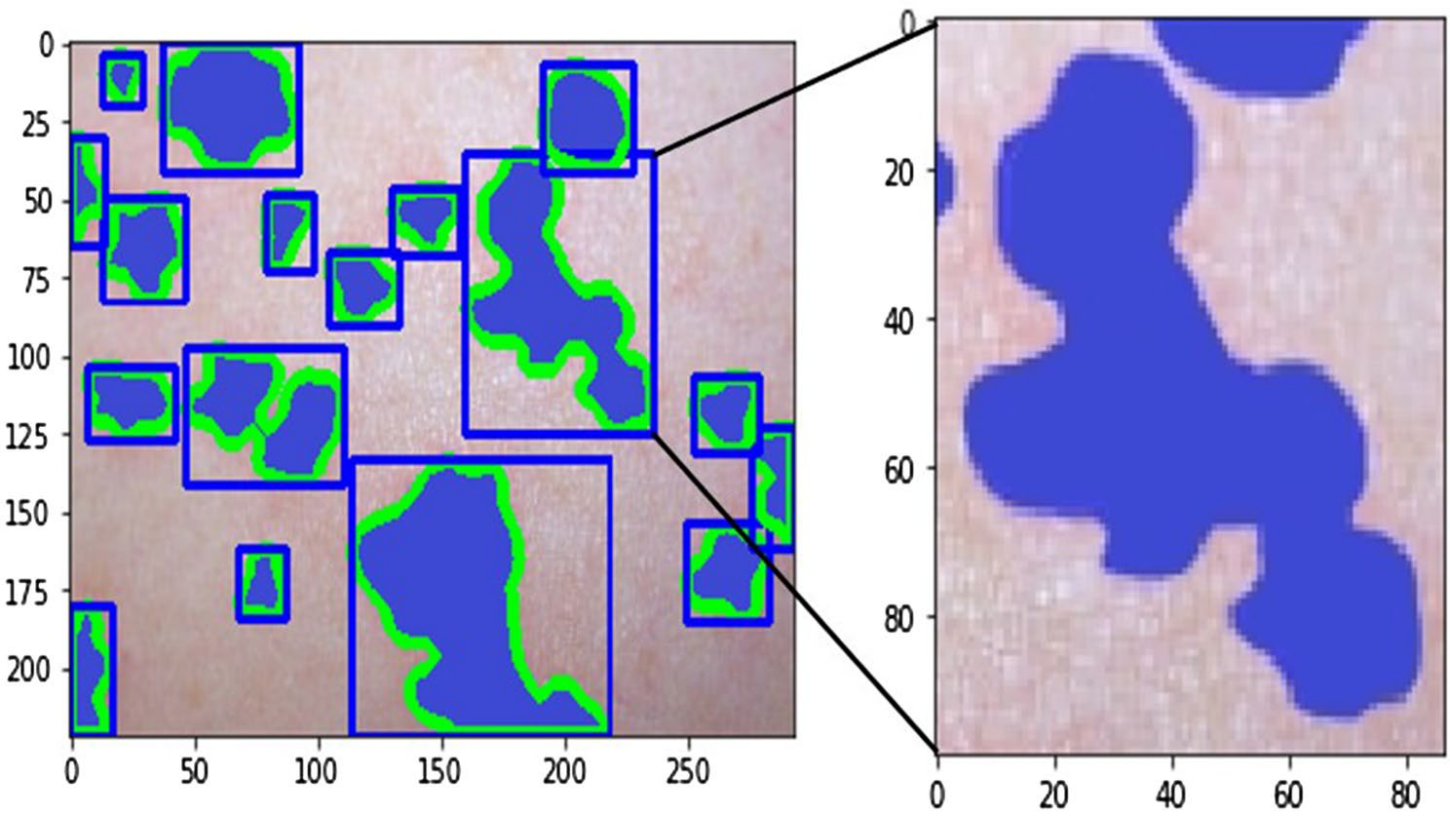
Contour finding algorithm applied to output of U-Net. Clusters of abnormal skin are identified through a contour finding algorithm. Each cluster is cropped in the shape of a rectangle through a convex hull algorithm used to surround each contour.

Using the decomposed images, in one instance, we input three images, namely, the original, the hemoglobin, and the melanin images, to our U-Net and obtained a single black-and-white mask image as output as shown in Fig. 8. In this image, a black pixel represented normal skin, and a white pixel represented abnormal skin. Using the mask image, we used a simple contour-finding algorithm to draw an outline around clusters of erythema. We then used a convex hull algorithm to draw rectangles around the contours. The dimensions and locations of these rectangles were then used to crop the original image. These cropped images of each cluster were saved as individual pictures. We added padding to each cluster to create a larger and squarer image, as the performance of classification can suffer due to clusters being too small or not evenly shaped. Figure 9 shows contours and rectangles around each cluster showing how each cluster was cropped.

After generating three replicas of our dataset, we create a unique 90-to-10 training and validation set. With each replica, we perform a grid search algorithm to find the optimal hyperparameters. For the loss function, we test the *Binary Cross-Entropy* and *Dice Coefficient Loss*. For the optimizer, we test *Adam* with learning rates of 1e−4, 5e−5, and 1e−5; *RMSprop* with learning rates of 1e−4, 5e−5, and 1e−5; and *SGD* with a momentum of 0.9 and learning rates of 1e−1, 5e−2, and 5e−2. For the number of epochs, we test with 40, 60, and 80 epochs and decrease the learning rate by a factor of 0.1 every 20 epochs. After testing with the replicas, we use the full training set for training with the hyperparameters: *Binary Cross-Entropy, Adam* with a learning rate of 5e−4, a weight decay of 5e−4, 60 epochs, and a decrease in learning rate by a factor of 0.1 every 20 epochs.

As our main objective was to demonstrate the viability of CAD, the performance was mostly determined using pixel-level sensitivity rather than the Intersection over Union or the Dice coefficient metrics that are often used to measure segmentation performance. Moreover, we mainly focused on the true positive rates of segmentation, represented by the sensitivity metric. This is because our aim was to create a screening test method to help healthcare workers make a more accurate diagnosis by preventing abnormal skin from being overlooked. Nevertheless, we also measured the performance of our model using the specificity, Dice coefficient, and Hausdorff distance to provide a more complete performance comparison. We measured these metrics by comparing the output from our U-Net model to an image that was masked by professional dermatologists. Going through each pixel, if a pixel of the U-Net output was black and the pixel of the dermatologist-masked image at the same location was black, this is seen as a true negative. If both were white, this was seen as a true positive. If the U-Net output was black but the dermatologist mask was white, this was seen as a false negative, and the converse was a false positive. The equations for sensitivity, specificity, and Dice coefficient metric can be represented by the following formulas:

$$Sensitivity= \frac{TP}{TP+FN}$$

$$Specificity= \frac{ TN}{TN+FP}$$

$$Dice Coef.= \frac{2 \times TP}{\left(TP+FP\right)+(TP+FN)}$$

The Hausdorff distance (HD) is used to measure the dissimilarity between the predicted segmentation masks the and ground truth. The Hausdorff distance can be calculated by the formula^18^:

$$Set X= \left\{{x}_{1}, \dots {x}_{n}\right\} and Y=\{{y}_{1},\dots , {y}_{n}\}$$

$$H\left(X,Y\right)=\mathrm{max}\left(h\left(X,Y\right),h\left(Y,X\right)\right),$$

where $$h\left(X,Y\right)=\underset{x\in X}{\mathrm{max}}\underset{y\in Y}{\mathrm{min}}\Vert x-y\Vert.$$

We use an implementation of the method presented^18^ to calculate the Hausdorff distance between the output and ground truth.

## Classification

EfficientNets^18^ were introduced in late 2019 as a state-of-the-art model for image classification. Rather than scaling a CNN model without balance between the depth, width, and resolution of the image at hand, EfficientNets were developed by scaling a baseline model in a methodical manner. This allows for an efficient increase in accuracy rates without unreasonable amounts of required memory and floating-point operations (FLOPS) through the optimization of the following formulas^18^:

$$\underset{d,w,r}{\mathrm{max}}Accuracy(N(d,w,r))$$

$$\mathrm{such \,that}: N\left(d,w,r\right)= \underset{i=1\dots s}{\odot }{\widehat{\mathcal{F}}}_{i}^{d\bullet \widehat{{L}_{i}}}({X}_{<r\bullet {\widehat{H}}_{i},r\bullet {\widehat{W}}_{i}, w\bullet {\widehat{C}}_{i}>})$$

$$Memory\left(N\right)\le target memory$$

$$FLOPS\left(N\right)\le target flops$$

Here, *d*, *w*, and *r* represent the depth, width, and resolution of the scaled model, and $\widehat{H}, \widehat{W},\widehat{C},\widehat{\mathcal{F}},\widehat{L}$ represent the parameters of the optimized baseline model. Thus, in summary, the goal of the EfficientNet model, namely, $N(d,w,r)$, is to produce maximum accuracy in a classification problem. The model is represented by the product of its variable-weighted parameters, represented as $\underset{i=1\dots s}{\odot }{\widehat{\mathcal{F}}}_{i}^{d\bullet \widehat{{L}_{i}}}({X}_{<r\bullet {\widehat{H}}_{i},r\bullet {\widehat{W}}_{i}, w\bullet {\widehat{C}}_{i}>})$. The memory usage, $Memory\left(N\right)$, and required computational performance, $FLOPS\left(N\right)$, for the model must be less than that of the target.

The original paper^19^ presents eight different models, ranging from EfficientNet-B0 through EfficientNet-B7, each increasing in complexity. Table 8 shows the accuracy and training time per epoch of each of these models trained on unsegmented images. There are sharp increases in training time between the EfficientNet-B4 and EfficientNet-B7 models, as we were forced to use smaller batch sizes during training owing to the increased number of trainable parameters and the limited memory in our GPU. In addition, as we employ a grid search algorithm, many models must be trained for many epochs. Therefore, a lower training time is desirable. After testing these models with our dataset and hardware, we chose to implement the EfficientNet-B4 model as it used substantial memory and training time without losing excessive complexity. We applied transfer learning to the segmented and cropped images from the previous section and classified them into 18 different classes.

**Table 8:** Training time required for efficientnet-B0 through B7.

Model	Top-1 accuracy (%)	Training time per epoch (s)
EfficientNet-B0	39.71	187.965
EfficientNet-B1	43.15	250.170
EfficientNet-B2	44.46	255.180
EfficientNet-B3	43.30	309.375
**EfficientNet-B4**	**45.77**	**392.925**
EfficientNet-B5	45.54	522.975
EfficientNet-B6	45.83	643.965
EfficientNet-B7	47.54	942.720

We further improved the performance by using the Synthetic Minority Oversampling Technique^20^ library, as a more balanced dataset was needed for training. In addition, because our segmentation model required more data to better generalize erythema, there were clusters of normal skin that were cropped and included in different classes. It was observed that this confused the model, as similar images were seen throughout different classes. To combat this, we refined the data by going through each image and excluding certain images that were either too small or incorrectly segmented images.

We created replicas of the training set and performed a grid search algorithm, as in the method utilized in the segmentation phase. For the loss function, we tested the *Categorical Cross-Entropy* and *Focal Loss*. For the optimizer, we test *Adam* with learning rates of 1e−4, 5e−5, and 1e−5; *RMSprop* with learning rates of 1e−4, 5e−5, and 1e−5; and *SGD* with a momentum of 0.9 and learning rates of 1e−1, 5e−2, and 5e−2. For the number of epochs, we test with 40 epochs, 60 epochs, and 80 epochs and decrease the learning rate by a factor of 0.1 every 20 epochs. After testing with the replicas, we used the full training set for training with the hyperparameters: *Categorical Cross-Entropy, Adam* with a learning rate of 1e−5, a weight decay of 5e−4, 80 epochs, and a decrease in learning rate by a factor of 0.1 every 20 epochs. The AUC is calculated by taking the integral of the curve created by points at different sensitivity and specificity thresholds. In addition, specificity, sensitivity, and the F1-score can be represented by the following formulas:

$$Specificity= \frac{ TN}{TN+FP}$$

$$Sensitivity= \frac{TP}{TP+FN}$$

$$F1-score= \frac{2TP}{2TP+FP+FN}$$

For all performance metrics, scores are calculated individually for each class present in the dataset. The scores are then weighted and averaged according to the number of data points in a class corresponding to the entire dataset.

### Ethics declarations

This study was exempted from the approval by the Institutional Review Board of Seoul National University Boramae Medical Center (No. 07-2020-148). The informed consent was waived by the Institutional Review Board of Seoul National University Boramae Medical Center because patient records/information was anonymized and de-identified prior to analysis. All experiments were performed in accordance with the relevant guidelines and regulations.

## Conclusion

We have shown that even without a large dataset and high-quality images, it is possible to achieve sufficient accuracy rates. In addition, we have shown that current state-of-the-art CNN models can outperform models created by previous research, through proper data preprocessing, self-supervised learning, transfer learning, and special CNN architecture techniques. Furthermore, with accurate segmentation, we gain knowledge of the location of the disease, which is useful in the preprocessing of data used in classification, as it allows the CNN model to focus on the area of interest. Lastly, unlike previous studies, our method provides a solution to classify multiple diseases within a single image. With higher quality and a larger quantity of data, it will be viable to use state-of-the-art models to enable the use of CAD in the field of dermatology.

## Data Availability

The dataset used for segmentation is available upon request from the corresponding author for academic use. The dataset used for classification is available on Dermnet for academic use (https://dermnetnz.org/).

## Code availability

The code for the development of a mobile app is available on (https://github.com/sonhamin/dermtools). The code regarding the analysis model is available upon request from the corresponding author for academic use.
